# Successful Treatment of Disseminated Carcinomatosis of the Bone Marrow Secondary to Colorectal Cancer With Modified FOLFOX6 Plus Panitumumab: A Case Report

**DOI:** 10.7759/cureus.91622

**Published:** 2025-09-04

**Authors:** Junsei Noda, Akinori Sasaki, Risa Okamoto

**Affiliations:** 1 Gastroenterology, Tokyo Bay Urayasu Ichikawa Medical Center, Urayasu, JPN

**Keywords:** colorectal cancer, disseminated carcinomatosis of the bone marrow, disseminated intravascular coagulation, folfox, panitumumab

## Abstract

Disseminated carcinomatosis of the bone marrow (DCBM) is a rare, aggressive manifestation of colorectal cancer (CRC). It is often accompanied by pancytopenia and disseminated intravascular coagulation (DIC) and has a poor prognosis. Herein, we present the case of a 61-year-old male patient with sigmoid colon cancer and DCBM who presented with fever, fatigue, and DIC. Bone marrow biopsy confirmed poorly differentiated adenocarcinoma, consistent with the primary tumor. Genetic testing revealed the RAS and BRAF wild-type status. The patient was administered modified FOLFOX6 plus panitumumab as first-line chemotherapy. The patient’s clinical symptoms improved rapidly, and transfusion dependency resolved after two cycles of chemotherapy. Subsequent comprehensive genomic profiling identified a subclonal *NRAS* G12D mutation; however, panitumumab treatment was continued because it was initially effective. Computed tomography performed two months later showed a marked reduction in liver metastases, and the patient sustained disease control since May 2025. To our knowledge, there are no previous reports of end-organ improvement and sustained response to chemotherapy containing an anti-EGFR antibody in a patient with CRC complicated by DCBM and DIC. This case highlights the potential benefits of early and appropriate therapy even in critically ill patients with rare metastatic patterns.

## Introduction

Colorectal cancer (CRC) is the third most common cancer and the second leading cause of cancer-related deaths worldwide [1]. Chemotherapy regimens including oxaliplatin, irinotecan, and fluoropyrimidine remain the standard treatments for unresectable CRC [2,3]. The prognosis for unresectable CRC has improved to approximately 2-3 years with the development of various chemotherapy regimens [4]. In particular, patients with left-sided CRC and RAS and BRAF wild-type in genetic testing can be treated with anti-EGFR antibodies, which are expected to have a high treatment response and improved progression-free and overall survival [5]. In the advanced stages of malignancies, clinicians should remain vigilant for rare manifestations such as disseminated carcinomatosis of the bone marrow (DCBM), as early recognition can be crucial for timely and appropriate management.

The most common site of CRC metastasis is the liver; however, it can spread to the lungs and peritoneum [4]. CRC is rarely accompanied by DCBM. CRC patients with DCBM have a high incidence of pancytopenia and disseminated intravascular coagulation (DIC) and an extremely poor prognosis [6]. In addition, the efficacy and safety of chemotherapy in patients with CRC accompanied by DCBM remain unclear.

Herein, we report a case of DCBM with DIC as the first symptom of unresectable CRC that was successfully treated with modified FOLFOX6 (mFOLFOX6) plus panitumumab. The patient provided informed consent for the presentation of anonymized clinical information.

## Case presentation

**Figure 1:**
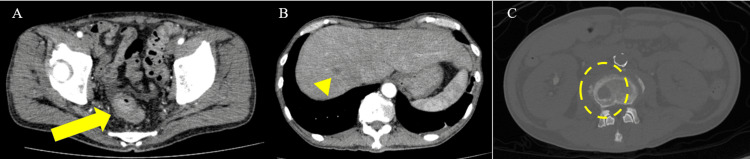
Abdominal computed tomography (A-C) Abdominal computed tomography shows an unevenly strengthened wall of the upper rectum (arrow) and liver (arrowhead) and bone (dashed circle) metastases

**Figure 2:**
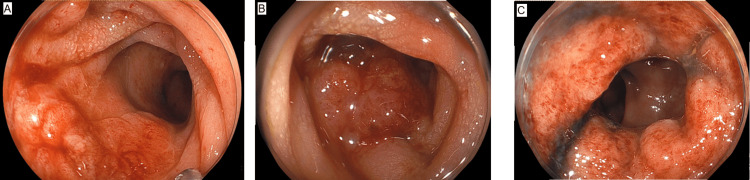
Lower gastrointestinal endoscopy (A-C) Lower gastrointestinal endoscopy reveals Borrmann type 3 colorectal carcinoma located 10–15 cm from the anal verge in the upper rectum

A 61-year-old male patient presenting with fever and fatigue for several weeks was referred to our hospital. The patient had a history of diabetes mellitus. Laboratory tests revealed bicytopenia (hemoglobin, 6.3 g/dL; platelet count, 48,000/μL), coagulation disorders (prothrombin time, 15.9 seconds; fibrinogen degradation product, 132.6 μg/mL), and elevated levels of carcinoembryonic antigen (118.9 ng/mL; reference range: <5.0 ng/mL) and carbohydrate antigen 19-9 (56.1 ng/mL; reference range: <37 U/mL). Computed tomography (CT) revealed an unevenly strengthened wall in the sigmoid colon and liver and osteolytic bone metastases (Figure 1). Colonoscopy revealed Borrmann type 3 cancer in the sigmoid colon. A biopsy of the primary tumor revealed poorly differentiated adenocarcinoma (Figure 2). In addition, a bone marrow biopsy was performed to investigate the cause of bicytopenia. Pathological examination of the bone marrow revealed adenocarcinoma, and immunostaining results were consistent with those of the primary tumor.

**Figure 3:**
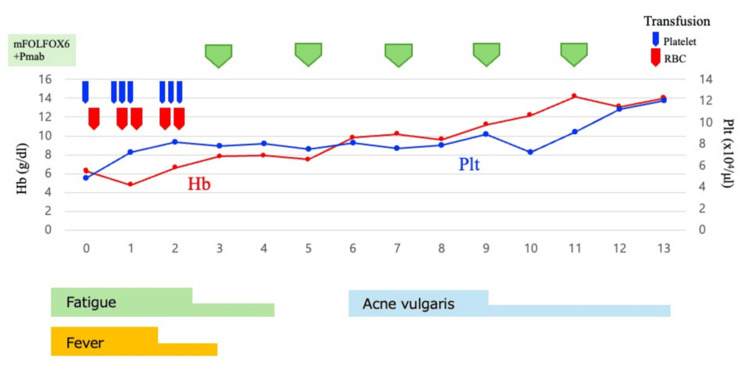
Timeline of chemotherapy, transfusions, and clinical symptoms Hemoglobin (Hb) and platelet (Plt) levels over 13 weeks of chemotherapy with mFOLFOX6 plus panitumumab. Transfusion events (red for RBC, blue for platelet) were limited to the early phase of treatment. Symptoms, including fatigue, fever, and acneiform rash, are also shown. Notably, no transfusions were required after week 2, indicating hematologic improvement following therapy initiation. RBC: red blood cell

**Figure 4:**
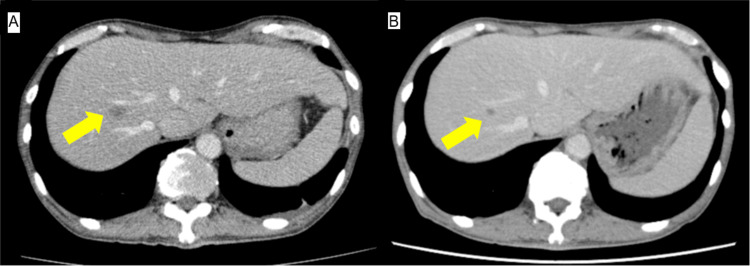
Abdominal computed tomography (A, B) Abdominal computed tomography reveals shrinkage of the known liver metastasis (arrow), and no new metastasis was found.

Based on these findings, the patient was diagnosed with unresectable CRC and DCBM with DIC. Subsequently, the following therapy-related target genes were detected: RAS was negative; BRAF was negative; MLH1, MSH2, MSH6, and PMS2 were positive; HER2 was negative. The patient received first-line chemotherapy with an mFOLFOX6 plus panitumumab regimen at reduced doses every two weeks: oxaliplatin (65 mg/m^2^), leucovorin (400 mg/m^2^), continuous infusion of 5-fluorouracil (2,400 mg/m^2^ over 46 hours), and panitumumab (6 mg/kg). The patient initially required both red blood cell and platelet transfusions due to bicytopenia at the start of chemotherapy; however, transfusions were no longer necessary after completing two courses. Furthermore, within 2-3 weeks of treatment initiation, the patient’s general condition began to gradually improve, accompanied by a reduction in fever and fatigue (Figure 3). The patient had grade 1 acne vulgaris, most likely attributable to panitumumab, as anti-EGFR agents are known to cause dermatologic toxicity. The rash was well-tolerated with the use of a topical steroid. Subsequently, multigene panel-based comprehensive genomic profiling (CGP) using FoundationOne CDx (Foundation Medicine, Cambridge, MA, US) detected alterations in NRAS G12D (subclonal), APC, and SOX9. Although initial molecular testing (RAS/BRAF status) was conducted prior to treatment initiation, we performed CGP during first-line chemotherapy in accordance with Japanese guidelines, which recommend early CGP testing in patients with unresectable advanced cancer. Given the presence of DCBM, which is associated with a particularly poor prognosis, early CGP testing was considered especially important for exploring potential therapeutic options. The NRAS mutation was first identified through this CGP analysis. The therapeutic strategy was discussed by our intrainstitutional molecular tumor board (expert panel). At the time the subclonal NRAS mutation was identified, treatment with mFOLFOX6 plus panitumumab was already achieving a favorable clinical response. Considering that the NRAS mutation was subclonal and its clinical significance for anti-EGFR resistance was uncertain, the panel recommended continuing panitumumab in combination with chemotherapy. CT performed two months after treatment initiation revealed shrinkage of the liver metastasis, with no new metastatic sites (Figure 4). As of May 2025, the patient continued receiving mFOLFOX6 plus panitumumab without tumor progression.

## Discussion

Herein, we report the case of DCBM with DIC as the first symptom of unresectable CRC that was treated with mFOLFOX6 plus panitumumab. Chemotherapy was effective, and pancytopenia improved, allowing the patient to be discharged. However, the efficacy of chemotherapy in patients with unresectable CRC with DCBM remains unclear. In general, these patients develop DIC at a high rate, making it impossible to administer chemotherapy. To the best of our knowledge, there are no previous reports describing an exceptional response to mFOLFOX6 plus panitumumab in a CRC patient with DCBM and DIC.

DCBM is characterized by widespread organ infiltration, including the bone marrow, and it is associated with a high incidence of DIC, resulting in an extremely poor prognosis [7]. The main symptoms of DCBM include anemia, back pain, and bleeding tendency [8]. Erythroblastic anemia, microangiopathic hemolytic anemia, elevated levels of alkaline phosphatase or lactate dehydrogenase, and DIC occasionally occur. Gastric cancer is the most common primary cancer that causes DCBM, followed by prostate and breast cancers. However, CRC accompanied by DCBM is rare [9]. The histological types of carcinomas that cause DCBM include diffuse types such as signet ring cell carcinoma and poorly differentiated adenocarcinoma; additionally, our case showed poorly differentiated adenocarcinoma on histological examination [10,11].

A review of case reports of patients with CRC complicated by DCBM who underwent chemotherapy is shown in Table 1. These cases were obtained through a PubMed search using the following search terms: “Disseminated carcinomatosis of the bone marrow” and “rectum” and “chemotherapy,” “Disseminated carcinomatosis of the bone marrow” and “rectal” and “chemotherapy,” or “Disseminated carcinomatosis of the bone marrow” and “colon” and “chemotherapy.” Nine patients with CRC complicated by DCBM, including our patient, were treated with chemotherapy [8,12-18]. The median age was 65 years (range, 51-79 years), and eight patients (88.9%) were male. The primary lesion was in the rectum in five cases, the sigmoid colon in three cases, and the right colon in one case, which was the transverse colon. The concomitant metastases at the time of DCBM diagnosis were mostly lymph node metastases (n = 5), with some patients showing liver metastases (n = 2). In contrast, two patients presented with DCBM at the time of diagnosis. Chemotherapy with fluoropyrimidine and oxaliplatin combination therapy was commonly used (n = 7); however, this is the only case in which mFOLFOX6 plus panitumumab therapy was administered based on genetic testing. The median progression-free survival in first-line chemotherapy was 4.0 months (95% confidence interval, 1.2-6.8), and the median overall survival was 9.4 months (95% confidence interval, 6.5-12.3) in patients in this literature review. Our patient showed progression-free survival of ≥7.5 months with first-line chemotherapy, which is a favorable result compared with previous cases.

**Table 1:** Reported cases of colorectal cancer (CRC) with disseminated carcinomatosis of the bone marrow treated with chemotherapy PFS: progression-free survival; OS: overall survival

Reference	Age	Sex	Primary site	Histology	Other metastatic lesions	Genetic mutation	Chemo regimen	PFS (month)	OS (month)
Huang et al., 2005 [12]	79	Male	Rectum	mod	None	Not examined	5-FU, LV	2.5	2.8
Naito et al., 2014 [13]	61	Male	Transverse colon	sig	Lymph node	Not examined	CAPOX + Bev	No data	No data
Nakashima et al., 2014 [14]	65	Male	Rectum	muc	Lymph node	Not examined	CAPOX	3.0	4.3
van Bunderen et al., 2014 [15]	65	Female	Sigmoid colon	sig	Lymph node	Not examined	CAPOX	4.0	7.0
Hanamura et al., 2016 [16]	60	Male	Sigmoid colon	por	None	RAS:WT, BRAF:WT	mFOLFOX6	5.0	10.0
Takeyama et al., 2017 [17]	65	Male	Rectum	mod	Lung	Not examined	mFOLFOX6	6.0	8.8
Nakamura et al., 2017 [18]	51	Male	Rectum	por	Liver, pararenal gland, peritoneum, and pleura	RAS:WT	5-FU/LV	1.0	22.7
Yoshida et al., 2021 [8]	66	Male	Rectum	por	Lymph node	RAS:WT	mFOLFOX6	5.0	11.1
Present case, 2024	61	Male	Sigmoid colon	por	Lymph node, liver	KRAS:WT, NRAS (subclonal), BRAF:WT, HER2: 1+, MSS	mFOLFOX6 plus panitumumab	7.5	Not reached

In addition, multigene panel-based CGP was performed to investigate >300 genetic alterations in this study. NRAS G12D subclonal mutations and alterations in APC and SOX9 were detected using CGP. No studies have investigated the genetic alterations in CRC-related DCBM, but there is one previous study on breast cancer [19]. The study suggested an association between PIK3CA mutations plus the overexpression of CCNE1 and MYC genes and DCBM. By accumulating cases of DCBM in CRC and analyzing oncogenes, it may be possible to identify the causative genes of DCBM in CRC and breast cancer. In contrast, molecular profiling, including that of oncogenes, differs between primary tumors and bone marrow metastases [19]. Moreover, oncogenes and the tumor microenvironment, including myeloid-derived suppressor cells and regulatory T cells, are associated with DCBM [20]. Therefore, in addition to conventional cancer genome profiling tests, a multifaceted approach using single-cell and multiomics analyses is necessary in the future.

## Conclusions

To our knowledge, this is the first report of an exceptional response to mFOLFOX6 plus panitumumab in a CRC patient with DCBM and DIC. Although patients with CRC complicated by DCBM generally have a poor prognosis, it is demonstrated that appropriate chemotherapy can lead to disease control even in severe cases. Our case further highlights the potential benefit of initiating anti-EGFR therapy in RAS wild-type patients before CGP results are available, based on initial RAS/BRAF testing. Further studies are warranted to elucidate the pathophysiology of DCBM and to develop new therapeutic strategies using multigene panel-based CGP and single-cell analysis.
